# Gender-Specific Variation in the Prognosis of Papillary Thyroid Cancer TNM Stages II to IV

**DOI:** 10.1155/2012/379097

**Published:** 2012-12-06

**Authors:** Sheng-Hwu Hsieh, Szu-Tah Chen, Chuen Hsueh, Tzu-Chieh Chao, Jen-Der Lin

**Affiliations:** ^1^Division of Endocrinology and Metabolism, Department of Internal Medicine, Chang Gung Memorial Hospital, College of Medicine, Chang Gung University, Linkou 333, Taiwan; ^2^Department of Pathology, Chang Gung Memorial Hospital, College of Medicine, Chang Gung University, Linkou 333, Taiwan; ^3^Department of General Surgery, Chang Gung Memorial Hospital, College of Medicine, Chang Gung University, Linkou 333, Taiwan

## Abstract

To investigate the correlation between gender and the clinical presentation of papillary thyroid cancer and the long-term followup results, 435 patients who underwent total or near-total thyroidectomy were enrolled in this study. Among these papillary thyroid cancer patients, 12.2% showed lymph node metastases and a higher incidence of male patients in the N1b group. There were 65 from 316 female (20.6%) and 49 from 120 male (40.8%) patients who had a postoperative disease progression. A total of 55 (12.6%) patients died of thyroid cancer. Male patients showed a higher thyroid cancer mortality than the females. Multiple regression analysis showed that male gender was an independent risk factor for cancer recurrence and mortality. Male patients with TNM stages II to IV of papillary thyroid cancer need to adopt aggressive surgical and postoperative ^131^I therapy.

## 1. Introduction

Thyroid nodules and cancer have long been considered to occur predominantly among women [1]. On the other hand, male gender has been reported to be an important risk factor for the development of the subgroup of well-differentiated thyroid cancers, based on the occurrence of larger tumors [2], worse prognosis in follicular and Hürthle cell thyroid cancers [3], and lymph node invasion and local neck recurrence of papillary thyroid carcinomas [4–6]. In contrast, no gender-specific differences have been observed in surgically treatable Graves' disease and toxic thyroid nodules [7]. Thyroid cancer is associated with a wide range of prognoses with different histopathologic patterns. In addition, other factors, such as TNM stage, surgical method, and the application of postoperative adjuvant therapy, may also influence therapeutic outcomes. Even in papillary thyroid carcinoma, different histologic variants with variable presentation and prognoses after treatment have been reported [8].

This study aimed to determine the role of gender in the clinical presentation of papillary thyroid cancer and in the results of long-term followup schemes. To avoid bias due to the influence of age and the less aggressive nature of this cancer in women, papillary thyroid cancers at TNM stage I were excluded from the study. All enrolled patients underwent total thyroidectomy and postoperative remnant ablation. Long-term data were analyzed to identify trends in thyroid cancer progression in relation to gender and age groups.

## 2. Patients and Methods

All patients enrolled in this study underwent total or near-total thyroidectomy and postoperative ^131^I treatment for remnant ablation in Chang Gung Memorial Hospital (CGMH) in Linkou, Taiwan. A total of 435 patients, consisting of 315 women (mean age 56.6 ± 8.9 years) and 120 men (mean age 57.9 ± 9.1 years), meeting the inclusion criteria during the treatment period between 1986 and 2009 were included in this study. All patients underwent regular followup until the end of 2011. A pathologic review was performed for all thyroid carcinomas using the World Health Organization (WHO) classification [9].

After thyroid surgery, thyroid remnant ablation was recommended 4–6 weeks after surgery for patients with papillary thyroid cancers, as in our previous study [10]. The ^131^I ablation dose for most patients was 1.1 GBq (30 mCi). A whole-body scan (WBS) was performed 1 week after ^131^I administration by using a dual-head gamma camera (Dual Genesys, ADAC, USA) equipped with a high-energy collimator. Cases in which the foci of ^131^I uptake extended beyond the thyroid bed were classified as cases of persistent disease or metastases. These patients were given a higher therapeutic dose of 3.7–7.4 GBq (100–200 mCi) 3 to 6 months later. Hospital isolation was arranged for those who received doses exceeding 1.1 GBq, and a WBS was performed 2 weeks after administration of the higher therapeutic dose. 

All patients were staged according to the UICC-TNM criteria (6th edition) [11]. Patients showing disease progression after the operation were classified into a residual cancer group or a relapse group. Fine-needle aspiration cytology (FNAC), ^131^I WBS, or other noninvasive examination and elevated Tg levels were used to confirm the presence of local recurrence in the neck or distant metastases. The patients in the residual cancer group were diagnosed within a year of the first thyroidectomy, and those in the relapse group were diagnosed a year after the first thyroid surgery. At the end of 2010, patients were categorized as disease free if they showed negative results in the ^131^I WBS, undetectable Tg levels without thyroxine treatment and a TSH level ≥30 *μ*IU/mL, undetectable Tg antibody levels at the final followup, and no identifiable local or distant metastasis in the noninvasive examination. The study was carried out on humans in compliance with the Helsinki Declaration and following approval by ethics committee of the Institution Review Board in CGMH (reference number: 99-3565B).

All data are expressed as mean ± SE values. Univariate and multivariate analyses were performed to determine the significance of the various factors by using the Kaplan-Meier method and the log-rank test [12]. A *P* value < 0.05 was considered statistically significant. In addition, the survival rates were calculated using the Kaplan-Meier method and compared using the Breslow and Mantel-Cox tests.

## 3. Results

In the 435 subjects with papillary thyroid carcinoma, the mean age of the study population at enrollment was 57.0 ± 9.0 years. Male thyroid cancer patients were generally older than female patients, but no statistical difference was observed (57.9 ± 9.1 years versus 56.6 ± 8.9 years; *P* = 0.1934) (Table 1). Comparison of the mean tumor size in the two groups showed that tumors of a larger size were observed among males. In addition, the incidence of T3 and T4 based on the TNM classification was higher among males. Approximately 12.2% of the study population demonstrated pathologically proven lymph node metastases. There was a higher incidence of males in the N1b group, although no statistical significance was observed. Higher percentage of males was presented with distant metastases than females (10.8% versus 4.4%; *P* = 0.0136) at the time of diagnosis. Histological presentation at the time of thyroidectomy including TNM stage, multicentric pattern, postoperative Tg, and remnant ^131^I uptake percentage showed no differences between the genders.

During the followup period, 49 (40.8%) male and 65 (20.6%) female patients had residual or relapsed. Of this group, 52.6% (60 of 114) were diagnosed within the first year after thyroidectomy. Higher percentage of residual groups was diagnosed in males (28 of 49; 57.1%) than in females (32 of 65; 49.2%). Fifty-four patients (33 males and 21 females) had relapsed one year after the operation. The mean duration of relapse after the operation was 4.3 ± 0.4 years. A significantly higher relapse frequency was observed among male patients as compared to females (*P* = 0.0001). Approximately 27.8% of enrolled patients were classified as disease free during followup, with the male group showing a lower frequency than the females, although without statistical significance (*P* = 0.1267). Multiple regression analysis of clinical factors for postoperative progression showed that male gender, postoperative Tg and TNM stage are independent risk factors (Table 2).

**Figure 1:**
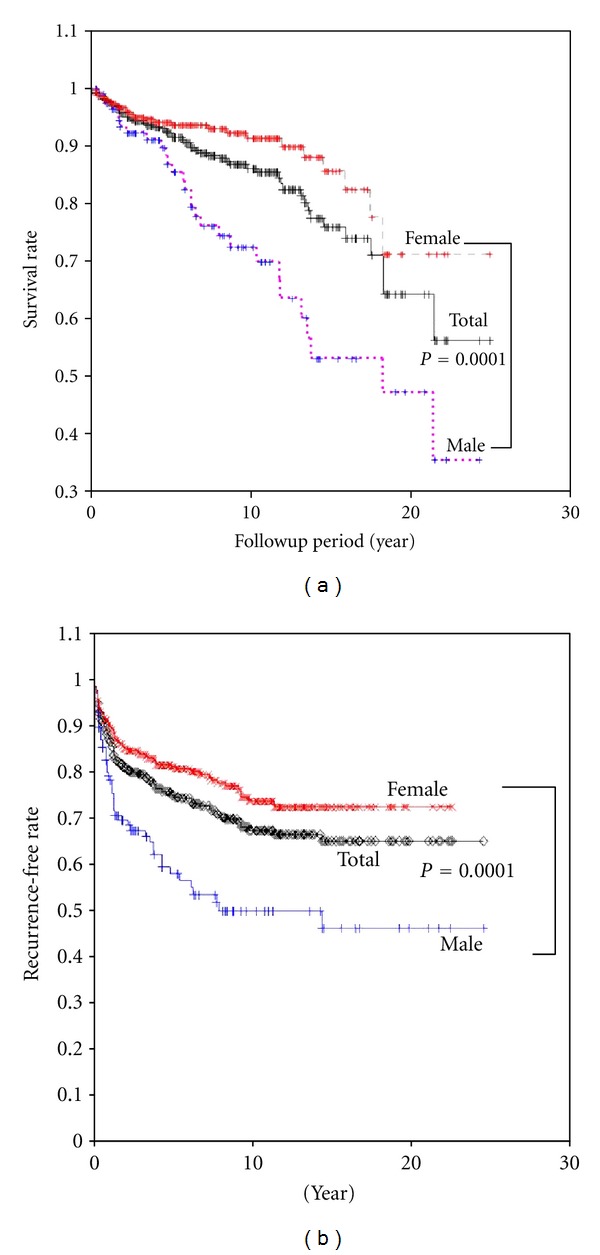
Thyroid cancer mortality (a) and recurrence-free survival (b) curves of the male, female, and total groups.

 After the mean followup period of 7.2 ± 0.3 years, 55 (12.6%) patients had died of thyroid cancer. The male population showed a higher thyroid cancer mortality than the female population (24.2% versus 8.3%; *P* = 0.0001).  Figure 1(a) shows the cancer-specific survival curves of the male, female, and total groups. The thyroid cancer-specific survival rates in the male, female, and total groups were 86.9%, 93.6%, and 92.3% at 5 years; 72.4%, 91.3%, and 86.1% at 10 years; and 47.2%, 71.2%, and 64.3% at 20 years, respectively. The recurrence-free rates for the male, female, and total groups are 58.9%, 81.5%, and 75.3% at 5 years; 50.1%, 73.9%, and 67.6% at 10 years; and 46.3%, 72.7%, and 65.2% at 20 years, respectively (Figure 1(b)). In addition, multiple regression analysis showed that male gender and TNM stage are independent risk factors in multiple regression analysis for thyroid cancer specific mortality (Table 3).

## 4. Discussion

The development of thyroid cancer involves multiple stages and genetic mutations, transforming normal follicular epithelial cells to differentiated malignant cancer cells [13]. During the processes of tumor initiation and progression, it has been reported that sex hormones may influence the rates of cancer cell proliferation, migration, or apoptotic change [14, 15].

According to prevalence studies, females have a higher incidence rate of thyroid nodules and surgical treatment than males [16, 17]. In contrast, the incidence of thyroid cancer in nodules is significantly higher in males. Because of differences in screening rates, gender-specific behavior, such as the greater tendency of men to seek medical attention later than females, and surgical methods, the therapeutic outcome varies between genders. [2, 6]. Previous study illustrated papillary thyroid cancer in females diagnosed at an earlier age than in males [18]. In contrast, there was no statistical difference of age between genders in this investigation. The main reason was the study exclude the patients in TNM stage I. Age has been used as an important prognostic factor in thyroid cancer, and this gender-specific variation thus makes survival analysis between the genders difficult. This study confirmed that based on a specific age group, TNM stage, and therapeutic modality, male gender is associated with a higher relapse rate and cancer-specific mortality.

Most patients with well-differentiated thyroid cancers have undergone long-term followup and treatment with good prognoses. It is important to minimize the risk of recurrence. Male gender has been reported to be an independent risk factor of cancer recurrence [5, 19]. In this study, consistent surgical and postoperative ^131^I therapy for papillary thyroid cancer in the same institute was associated with a higher recurrence rate. The frequency of male patients in the N1b group was shown to be higher than that of female patients. The incidence of micrometastases in the central neck region in patients staged as N0 by preoperative and intraoperative was recently assessed in papillary thyroid carcinoma [20]. Male gender was shown to be associated with lymph node micrometastasis. In addition, using univariate analysis, a significant correlation was shown between male gender and lung metastasis in papillary thyroid carcinoma presenting with bilateral lateral cervical lymph node metastasis [21]. In this study, prophylactic lymph node dissection was not performed on any of the patients. The role of prophylactic lymph node dissection or sentinel node biopsy among male patients at TNM stages II to IV need to be further investigated [22].

Recently published guidelines concerning high-risk cases of papillary and follicular thyroid carcinomas do not consider gender as a risk factor [23, 24]. More aggressive postoperative modalities, such as higher doses of ^131^I for thyroid remnant ablation and treatment for distant metastases, and closely monitored followup imaging after serum Tg level elevation need to be considered. Those with a histologic pattern of tall cell, insular pattern, and poorly differentiated thyroid cancer have a poorer prognosis, compared with those with classical, well-differentiated thyroid cancer [25, 26]. Our results illustrated that male patients had higher percentage of aggressive histologic patterns, otherwise no statistical difference. A number of limitations are also identified in this study. The data was selected from a single institution, and this is unlikely to represent the real prevalence of thyroid cancer in Taiwan. This study spanned more than 20 years, with different surgeons performing different surgical procedures. Similarly, the dose used in ^131^I therapy for papillary thyroid cancer varied among endocrinologists. In conclusion, this study demonstrated higher mortality and recurrence rates in males with papillary thyroid cancer in TNM stages II to IV, suggesting the need for more aggressive surgical treatment and postoperative ^131^I therapy for this specific group of patients.

## Figures and Tables

**Table 1:** Clinical features of papillary thyroid carcinoma by gender.

	Female (*n* = 315)	Male (*n* = 120)	Total (*n* = 435)	*P* value
Age	56.6 ± 8.9	57.9 ± 9.1	57.0 ± 9.0	0.1934
TNM stage (II/III/IV)	90/60/165	27/27/66	117/87/231	
T1*	128 (31.0%)	26 (19.0%)	154 (28.0%)	
T2	102 (24.7%)	35 (25.5%)	137 (24.9%)	0.4023
T3	39 (9.4%)	20 (14.6%)	59 (10.7%)	
T4	144 (34.9%)	56 (40.9%)	200 (36.4)	
Tumor size (cm)	3.0 ± 0.1	3.7 ± 0.2	3.2 ± 0.1	0.0001
LN metastases	38 (12.1%)	15 (12.5%)	53 (12.2%)	
N1a	13 (41.9%)	3 (21.4%)	16 (35.6%)	0.1834
N1b	18 (58.1%)	11 (78.6%)	29 (64.4%)	
Soft-tissue invasion	154 (48.9%)	51 (42.5%)	205 (47.1%)	0.2328
Distant metastases	14 (4.4%)	13 (10.8%)	27 (6.2%)	0.0136
Multicentric	92 (29.2%)	30 (25.0%)	122 (28.0%)	0.3827
Aggressive histological patterns^#^	8 (2.5%)	7 (5.8%)	15 (3.4%)	0.1366
^ 131^I dose accumulative dose (mCi)	152 ± 10.7	227 ± 27.9	173 ± 11.0	0.0023
Postoperative one month ^131^I uptake (%)	6.2 ± 0.8	4.9 ± 0.7	5.9 ± 0.6	0.3018
Postoperative progression	65 (20.6%)	49 (40.8%)	114 (26.2%)	0.0001
Relapse/Residual	33/32	21/28	54/60	0.4023
Period of relapse from diagnosis (year)	4.4 ± 0.7	4.1 ± 0.6	4.3 ± 0.4	0.7132
Disease free	94 (29.8%)	27 (22.5%)	121 (27.8%)	0.1267
Followup period (yr)	7.3 ± 0.3	6.8 ± 0.5	7.2 ± 0.3	0.3501
Cancer mortality	26 (8.3%)	29 (24.2%)	55 (12.6%)	0.0001

T1*: all the patients with local invasion or distant metastases.

Aggressive histological patterns^#^: tall cell, insular pattern, column cell, and poorly differentiated thyroid cancers.

**Table 2:** Multiple regression analysis for factors associated with postoperative progression.

	SE	Standardized coefficient	*t* value	*P* value	95% confidence interval	
					Lower	Upper
Intercept	0.2438	−0.677	−2.724	0.007	−1.167	−0.186
Age	0.004	0.10	1.533	0.127	−0.002	0.012
Followup period	0.005	−0.049	−0.711	0.478	−0.014	0.006
Postoperative one month ^131^I uptake	0.004	0.119	1.772	0.078	−0.001	0.014
Gender: female/male	0.068	0.251	3.672	0.000	0.115	0.383
Postoperative one month Tg	0.001	0.206	2.907	0.004	0.000	0.002
TNM stage	0.035	0.175	2.576	0.011	0.021	0.157

Dependent variable: postoperative progression (no/yes).

**Table 3:** Multiple regression analysis for factors associated with cancer mortality.

	Standardized coefficient	SE	*t *value	*P* value	95% confidence interval	
					Lower	Upper
Intercept	−0.594	0.194	−3.059	0.003	−0.978	−0.211
Age	0.104	0.003	1.405	0.162	−0.002	0.009
Followup period	−0.023	0.004	−0.322	0.748	−0.009	0.0067
Postoperative one month ^131^I uptake	0.055	0.003	0.784	0.434	−0.003	0.008
Gender: female/male	0.344	0.053	4.829	0.000	0.152	0.361
Postoperative one month Tg	−0.051	0.001	−0.688	0.434	0.000	0.001
TNM stage	0.140	0.027	1.98	0.049	0.002	0.107

Dependent variable: cancer mortality (yes/no).
